# Discontinuous Deformation Monitoring of Smart Aerospace Structures Based on Hybrid Reconstruction Strategy and Fiber Bragg Grating

**DOI:** 10.3390/s24113603

**Published:** 2024-06-03

**Authors:** Kangyu Chen, Hengzhen Fan, Hong Bao

**Affiliations:** 1 The School of Mechano-Electronic Engineering, Xidian University, Xi’an 710071, China; ky.chen@stu.xidian.edu.cn (K.C.); 22041212765@stu.xidian.edu.cn (H.F.); 2 Hangzhou Research Institute of Xidian University, Hangzhou 311231, China

**Keywords:** shape sensing, inverse finite element method, discontinuous structures, Fiber Bragg grating, geometric constraints

## Abstract

A hybrid enhanced inverse finite element method (E-iFEM) is proposed for real-time intelligent sensing of discontinuous aerospace structures. The method can improve the flight performance of intelligent aircrafts by feeding back the structural shape information to the control system. Initially, the presented algorithm combines rigid kinematics with the classical iFEM to discretize the aerospace structures into elastic parts and rigid parts, which will effectively overcome structural complexity due to fluctuating bending stiffness and a special aerodynamic section. Subsequently, the rigid parts provide geometric constraints for the iFEM in the shape reconstruction method. Meanwhile, utilizing the Fiber Bragg grating (FBG) strain sensor to obtain real-time strain information ensures lightweight and anti-interference of the monitoring system. Next, the strain data and the geometric constraints are processed by the iFEM for monitoring the full-field elastic deformation of the aerospace structures. The whole procedure can be interpreted as a piecewise sensing technology. Overall, the effectiveness and reliability of the proposed method are validated by employing a comprehensive numerical simulation and experiment.

## 1. Introduction

The trend for intelligent aircraft structures aims for efficient operation over a wide range of aerodynamic loads. Therefore, morphing wing technology has emerged as a highly promising option because of the capability of modifying the shape of the smart wing during flight to meet the demands of diverse and varied missions [1,2]. In contrast to the conventional fixed wing, the cornerstone of the morphing wing lies in the internal active control system that leverages the main bearing bone structure in tandem with adaptive skins to orchestrate wing deformation. Embedded within the aforementioned challenge is the reverse task of detecting or tracking structural movements instantaneously. This problem can be solved by developing a precise and dependable deformation sensing system tailored for aerospace structures [3].

The commonly utilized measurement techniques encompass photoelasticity, stereo vision, laser scanning, and coordinate measuring machines [4]. Nevertheless, the majority of these techniques are geared toward offline deformation monitoring in terrestrial environments and have limitations when it comes to real-time monitoring of aircraft structural deformation. With recent advancements in distributed strain sensing techniques like Fiber Bragg Grating (FBG) sensors, strain-based deformation sensing techniques have drawn considerable interest in aerospace; the strain sensors can fulfill the requirements for lightweight design, precision, and online monitoring in aerospace [5]. Meanwhile, optical frequency domain reflectometry (OFDR) can provide more comprehensive strain data across the entire structure and is also competitive in shape sensing [6,7,8].

The key to shape-sensing technologies using strain information is to establish the relationship between strain and displacement. Many methods have been proposed to deal with the issue [9]. Ko et al. obtained the structural deformation field by directly integrating the measured strain, named Ko’s displacement theory [10]. Foss et al. introduced the Modal Transformation Theory (MTT), which relies on matrices of strain mode and displacement mode [11]. Bruno uses a pure data-driven scheme to construct a neural network that maps the strain measured by the sensor directly to structural deformation [12]. These methods have been successfully applied in a lot of engineering structures like beams, trusses, and frame structures. However, these methods are seldom applied in complex structures, especially in real deformed wings. Due to a large number of training samples being required at the stage of constructing the network rules, especially when the model is complex, there will be a situation in which one measured strain corresponds to multiple deformations. It will bring difficulties to the shape sensing of morphing wings when it comes to a variety of external loads during flying services and complex geometries.

Compared with the deformation reconstruction algorithms mentioned above, the inverse finite element method (iFEM) has shown great potential because of its capability of full-field deformation reconstruction and independence of loading conditions and material properties [13]. The basic idea of the iFEM is to discretize the structure into a set of elements at first, like the finite element method (FEM). Then, the relationship between the element node displacement and element strains can be deduced based on the fundamental deformation theory [14]. After strains at the center of each element are monitored with sensors, the iFEM minimizes the error function between monitored strains and strains corresponding to node displacements. Consequently, the reconstruction capability of the iFEM highly depends on the partitioning of the inverse elements as well as the error function. At present, the class stiffness matrix during the deformation of the structure reconstructed from the strain is often determined by the pending parameters such as sensor measurement locations and distances, so the improper selection of the surface strain measurement points is the root cause of the element model pathology [15]. Zhao et al. used multi-objective optimization to assign the fiber grating location by taking the robustness and accuracy as optimization indexes [16].

Based on the idea of an inverse finite element, Gherlone et al. [17] developed an inverse beam element applicable to shear, bending, and torsional deformation, based on the theory of spatial Timoshenko beam deformation. Chen et al. [18,19] used the homogenized theory to improve the traditional iFEM, which can expand the application scope of inverse beam elements in engineering. Savino et al. [20] developed a method for reconstructing the displacement field of a circular arch structure based on the shape functions of two-node beam elements, and the simulation results demonstrated better reconstruction performance than traditional finite element analysis methods. Roy et al. [21] accurately predicted the torsional deformation of cantilevered airfoil beams by a proposed numerical function. Kefal et al. developed a shape function or a new solid element, which has good applicability to reconstructing curved structures [22,23,24]. Cerracchio et al. studied a three-node shell element shape function to reconstruct the reinforced plates [25]. Tabrizi et al. combined the iFEM with the smoothed element analysis to propose a new four-node plate-shell element. This method achieved the real-time reconstruction of the displacement field of a skewed woven wing-shaped laminated plate [26]. Esposito et al. [27] carried out deformation sensing research on a cantilever wing box with static flexural and torsional loading, and the wing box panel was partitioned into a large number of uniform rectangular elements. Niu et al. [28] applied the iFEM to a small antenna panel loaded with concentrated forces with the fixed end. Li et al. [29] explored the iFEM technique on a cryogenic composite tank under internal pressure, which was a cylindrical shell with a large structure size and complex form.

Up to the present, work on the iFEM has focused on the development of standard elements, specifically for the shape sensing of infrastructures with traditional cross-sections. As most prior investigations have simply and directly equated the wing to the plate or beam element, these sensing methods of aerospace structures have some limitations, which are mainly reflected in the need to place a large number of strain sensors to divide the element. Considering the small size of the stiffeners and the unsuitability of wiring, the sensor network of the monitoring system will be more complicated. Therefore, the main objective of this paper is to discuss and provide a novel reconstruction model for aerospace structures. The framework of this paper is organized as follows. In Section 2, the theoretical foundation and fundamental steps of the proposed reconstruction method are introduced. In Section 3, the engineering reliability of the proposed methodology, the E-iFEM, is substantiated by the comprehensive examination of the wing-integrated antenna structure subjected to different loading cases. Finally, some conclusions and the future direction of the reconstruction algorithm for the complex aerospace structures are summarized.

## 2. Theoretical Formulation of the Enhanced iFEM

**Figure 1:**
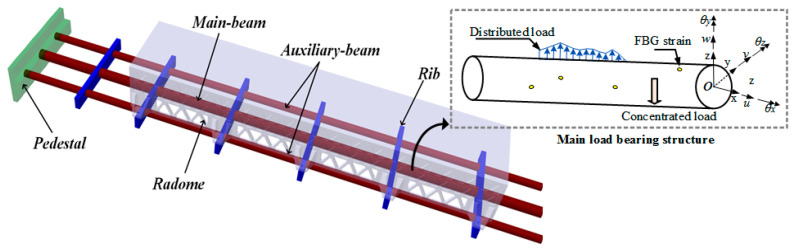
Geometric and planar sketch of the deformed wing.

For deformed wings embedded with conformal antennas, the primary load-bearing structures are positioned in parallel along the span direction and shown in Figure 1. Considering the connection between the conformal antenna and the stiffener, the support frame requires real-time shape sensing to provide feedback to the drive and control system. For each major load-bearing structure, only bending deformation occurs, so it can be simplified to Euler–Bernoulli beams constrained by the rigidness.

To further improve the efficiency and accuracy of the iFEM for complex structures, an enhanced iFEM based on the general iFEM method and piecewise discretization was proposed. Firstly, a suitable elastic inverse finite element and rigid component are selected to discretize the aerospace structures. Then, an error function of the measured strains and analytical strains is established, whose independent variable is the displacement of the inverse finite element node. The structural deformation can be determined by minimizing the weighted least squares of the functional. Finally, the full-field structural displacements can be acquired by supplementing the correct boundary constraints and assembling all the elastic-rigid elements. This method can discretize complex structures according to structural characteristics and effectively eliminate the reconstruction errors caused by discontinuous reinforcement structures.

### 2.1. Euler-Bernoulli Inverse Beam Formulation

Let us assume the smart wing is under a diverse loading condition, and the action of these loads only leads to bending deformation. Therefore, the inverse finite element model based on the kinematic hypothesis of the Euler–Bernoulli beam theory can be used to reconstruct the structure. In this context, the displacement vector can be expressed using **u** = {*w*,*θ_y_*}^T^.

(1) $$u_{x}(x,z)=z\theta_{y}(x)\;\;u_{z}(x,z)=w(x),$$

where *w*(*x*) is the mid-axis transverse displacements and *θ*_y_(*x*) represents the rotational degrees about *y*-axes. For small beam displacements, the axial and transverse shear strains can be determined as follows:

(2) $$\varepsilon_{x}=\frac{\partial u_{x}}{\partial x}=z\frac{\partial \theta_{y}}{\partial x}\;\;r_{xz}=\frac{\partial u_{x}}{\partial z}+\frac{\partial u_{z}}{\partial x}=\theta_{y}-\frac{\partial w}{\partial x},$$

Considering the interpolation of kinematic variables **u** within the element can be expressed by the shape functions. Hence, the two-node Hermite interpolation shape function was used to discrete the displacement and strain field. Therefore, the kinematic variables *w*(*x*) and *θ*_y_(*x*) can be expressed as follows:

(3) $$w(x)=N_{1}w_{1}+N_{2}\theta_{1}+N_{3}w_{2}+N_{4}\theta_{2}=N_{k}u^{e},$$

(4) $$\theta_{y}(x)=\frac{dw(x)}{dx}=B_{1}w_{1}+B_{2}\theta_{1}+B_{3}w_{2}+B_{4}\theta_{2}=(N_{k}{)}^{\prime }u^{e},$$

where **u***^e^* is the element node displacement array and can be abbreviated as follows:

(5) $$u^{e}={\left[w_{1}\;\theta_{1}\;w_{2}\;\theta_{2}\right]}^{T},$$

The Hermite polynomials *N*_1_, *N*_2_, *N*_3_, and *N*_4_ are the interpolation shape function, and the specific expressions can be written as follows:

(6) $$\begin{array}{cc} N_{1}=1-3{\left(\frac{x-x_{1}}{l}\right)}^{2}+2{\left(\frac{x-x_{1}}{l}\right)}^{3} & N_{2}=\left[\frac{x-x_{1}}{l}-2{\left(\frac{x-x_{1}}{l}\right)}^{2}+{\left(\frac{x-x_{1}}{l}\right)}^{3}\right]l\\ N_{3}=3{\left(\frac{x-x_{1}}{l}\right)}^{2}+2{\left(\frac{x-x_{1}}{l}\right)}^{3} & N_{4}=\left[{\left(\frac{x-x_{1}}{l}\right)}^{3}+{\left(\frac{x-x_{1}}{l}\right)}^{2}\right]l \end{array},$$

By combining Equations (3) and (4) with Equation (2), the discrete theoretical section strains are the following:

(7) $$\varepsilon_{x}=z\kappa (u^{e})=z(N_{k}{)}^{\prime \prime }u^{e}=zB_{k}u^{e}r_{xz}=0,$$

The strain matrix **B***_k_* can be calculated by substituting Equation (6) into Equation (7), and the expression is as follows:

(8) $$\begin{array}{cc} B_{1}=\frac{\partial^{2}N_{1}}{\partial x^{2}}=-\frac{6}{l^{2}}+\frac{12}{l^{3}}(x-x_{1}) & B_{2}=\frac{\partial^{2}N_{2}}{\partial x^{2}}=-\frac{4}{l}+\frac{6}{l^{2}}(x-x_{1})\;\\ B_{3}=\frac{\partial^{2}N_{3}}{\partial x^{2}}=\frac{6}{l^{2}}-\frac{12}{l^{3}}(x-x_{1}) & B_{4}=\frac{\partial^{2}N_{4}}{\partial x^{2}}=\frac{6}{l^{2}}(x-x_{1})-\frac{2}{l} \end{array},$$

According to the above calculation, the linear strain ε_x_ based on Hermite interpolation polynomial is represented. When the nodal degree of freedom (DOF) is determined in advance, the structural deformation field can be accurately described. Therefore, how to use a small amount of discrete strain to determine the nodal degree of freedom needs to be discussed.

### 2.2. Least-Squares Error Functional

**Figure 2:**
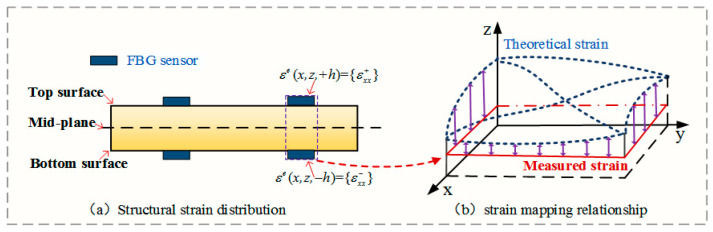
A discrete surface strain sensor distribution scheme.

For an Euler–Bernoulli beam, the strains of the upper and lower surfaces are anti-symmetric about the mid-plane, so the strain gauges need to be symmetrically pasted on the upper and lower surfaces of the beam element to obtain the accurate neutral axial strain. The specific strain sensor layout is shown in Figure 2. When the strain gauges are arranged on the surface of the beam element, the measured curvatures can be computed as follows:

(9) $$\kappa^{\varepsilon }=\frac{1}{2t}\left(\varepsilon_{xx}^{+}-\varepsilon_{xx}^{-}\right),$$

Considering the membrane strain and transverse shear strains can be omitted due to their values equal to zero, the least-squares error function on the node displacement between the curvatures *κ*(*u*^e^) and measured strains *κ*^ε^ can be simplified as follows:

(10) $$\Psi (u^{e})={\left\|\kappa \left(u^{e}\right)-\kappa^{\varepsilon }\right\|}^{2},$$

As the iFEM methodology is based on a variational formulation, the error functional for each element can be expressed using normalized Euclidean norms:

(11) $$\Psi (u^{e})=\frac{{\left(2t\right)}^{2}}{n}\int_{0}^{l}{\sum_{i=1}^{n}\left[\kappa {\left(u^{e}\right)}_{i}-\kappa_{i}^{\varepsilon }\right]}^{2}dx,$$

where *L* is the element length, and *N* is the number of axial beam sections. The analytical sectional strains are computed from the kinematic field of the structure, so the resolution of the elemental error function entails minimizing the said function concerning the nodal DOF.

(12) $$\frac{\partial \kappa (u^{e})}{\partial u^{e}}=ku-f=0,$$

Each component of the section strain is determined by the derivative matrix of the shape functions.

(13) $$k=\frac{L}{N}\sum_{i=1}^{n}\left[B_{k}^{T}(x)B_{k}(x)\right]\;\;f=\frac{L}{N}\sum_{i=1}^{n}\left[B_{k}^{T}(x)e^{\varepsilon }\right]$$

The system matrix **k** is only a function of strain sensor positions within the element, whereas the vector **f** is a function of the sensor positions and the experimental strain measurements. Hence, for every update in the strains, the displacements can be evaluated very computationally efficiently.

### 2.3. Enhanced Inverse Finite Element Method

By assembling the local reconstruction equation of each inverse finite element, the global set of equations for the reconstructed structure can be acquired. Additionally, the boundary condition, including geometric constraints and displacement constraints, is one of the key factors for the iFEM to accurately reconstruct the structural deformation.

**Figure 3:**
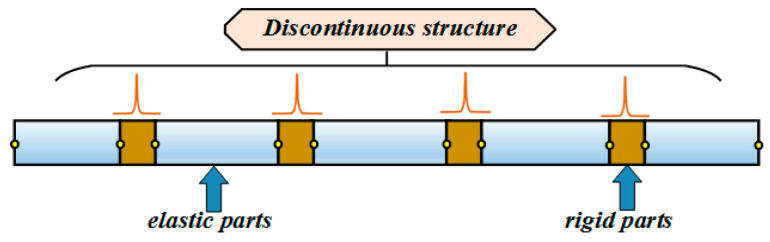
Analysis of discontinuous structures.

The proposed structure discretization method is suitable for the sensing of complex aerospace structures. According to the basic mechanical properties, the structure can be equivalent to the combination of two different parts, including the elastic part for reconstruction and the rigid part for boundary conditions, as shown in Figure 3.

**Figure 4:**
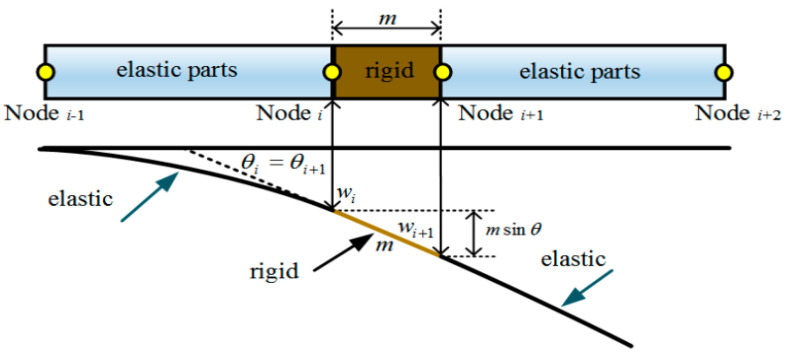
A specific description of the geometric relationship.

When the elastic part deforms, the rigid part only generates rigid displacement. Therefore, a geometric correlation between two adjacent elastic nodes has appeared; that is, the DOF of the initial node needs to be mapped to the following node, as shown in Figure 4. The DOF of a node *i* + 1 can be expressed by node *i* as follows:

(14) $$w_{i+1}=w_{i}+m\sin\theta_{i}\;\;\sin\theta_{i}\approx \theta_{i},$$

where *m* is the length of the rigid part region. Due to the magnitude of angle *θ_i_* being small, Equation (14) can be written as follows:

(15) $$\left\{\begin{array}{c} w_{i+1}\\ \theta_{i+1} \end{array}\right\}=\left[\begin{array}{cc} 1 & m\\ 0 & 1 \end{array}\right]\left\{\begin{array}{c} w_{i}\\ \theta_{i} \end{array}\right\},$$

Then, by using the necessary local coordinate transformation and combining all the element contributions, the global set of equations for the discontinuous elastic element was obtained as follows:

(16) KU=F

**Figure 5:**
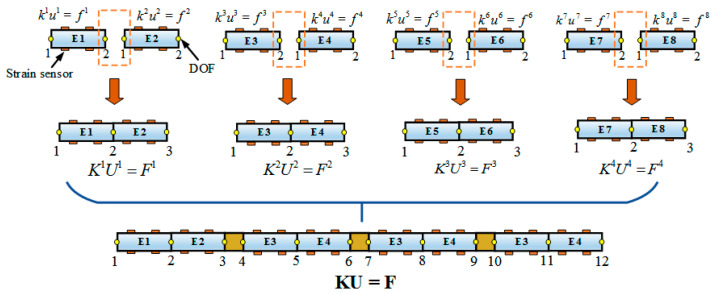
Visual description of the reconstruction model.

Similar to the FEM, the boundary condition is the key to ensure the non-singularity of the matrix. Hence, the difference between the obtained DOF by this scheme and the traditional iFEM will be discussed (see Figure 5).

In the traditional iFEM, **T^e^** only represents the node transformation matrix of DOF from a local to a global coordinate system. However, in the E-iFEM, **T^e^** was given additional geometric meanings by combining the geometric constraints of the rigid part.

(17) $$\begin{array}{c} K=\overset{N}{\underset{e=1}{\Omega }}[(T^{e}{)}^{T}k^{e}T^{e}]\;\;F=\overset{N}{\underset{e=1}{\Omega }}[(T^{e}{)}^{T}f^{e}]\\ U=\overset{N}{\underset{e=1}{\Omega }}[(T^{e}{)}^{T}u^{e}] \end{array}$$

**Figure 6:**
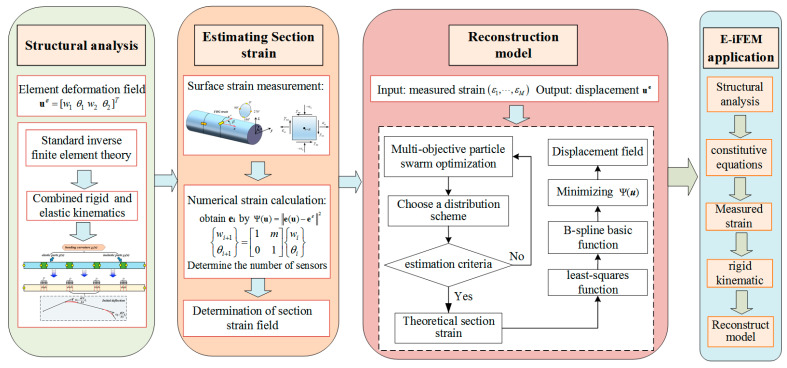
The flowchart of the E-iFEM.

Since the quasi-stiffness matrix K contains the rigid body motion of the discontinuous structure, it is necessary to add displacement boundary conditions Ω to build a transformation matrix connecting the local system to the global system. Similarly, the vector F can be determined online from the experimental strain and boundary DOF. The whole derivation process of the enhanced iFEM can be completed by the above-detailed description, and is shown in Figure 6.

Being aware of the flowchart that the inverse solution of the matrix exists in **U** = **K^−1^F**, the sensor layout algorithm is introduced to strengthen the robustness and reliability of the proposed reconstruction algorithm. In particular, the relevant MPSO algorithm has been described in great detail, and interested readers can refer to [15]. The sensor layout is conducted over the offline environment to eliminate the limited accuracy caused by the generalization input error. Therefore, the strain data can be collected by the measuring device in real time, so the online reconstruction of the structural deformation field is reliable.

## 3. Application

This section describes a practical application of the E-iFEM for the displacement reconstruction of different discontinuous aerospace structures using measurements from fiber-grating strain sensors. The experimental data are influenced by imperfections in the section shape and boundary conditions, and the comprehensive evaluation will be conducted on ABAQUS-6.14 and the ground experimental platform. Therefore, the work can provide valuable insight into the accuracy and reliability of the obtained E-iFEM results.

### 3.1. Simulation Study: Circular Section

In this section, experimental validation of the enhanced iFEM for the displacement reconstruction of the reinforced structure was performed using surface strain data. To analyze the influence of the complex geometric shape of the antenna frame on bending stiffness, the commercial software ABAQUS is used to establish a simulated example of the support platform. The model was composed of three circular beams of different thicknesses and six isothick ribs. The constructed finite element model can provide both discrete strain and detailed nodal displacement values as a reference. The former is input into the E-iFEM to reconstruct the node displacement and then interpolated to obtain the displacement field of the whole structure.

**Figure 7:**
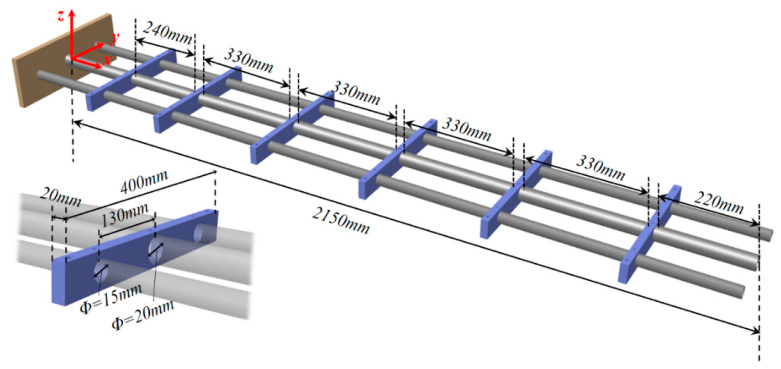
The geometric description of the airfoil frame.

**Figure 8:**
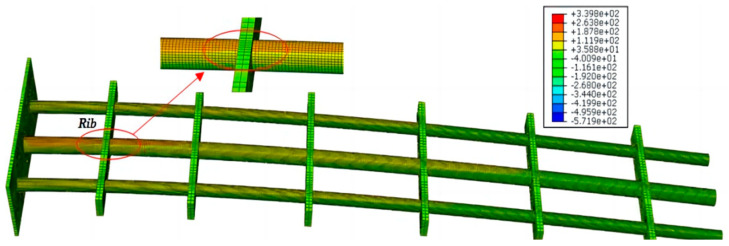
The visualizational stress cloud chart of the airfoil frame.

When setting up the structure and material layout, the isothick ribs are manufactured from structural steel, and the beams are manufactured from aluminum alloy. The ABAQUS model was modeled with 56,350 elements, including beam and solid elements. The geometric dimensions are shown in Figure 7, and the material properties of the modeled structure are introduced in Table 1. Since the fuselage connector will be fully constrained with the aircraft, the entire component can be considered a cantilever structure. Different static loading cases were applied in the simulation experiment, and the phenomenon of an abrupt change at the rib plate can be better observed in Figure 8.

Based on the commercial software calculation results, the deformed information, especially the strains and shear strains of collected nodes, can be used to implement the reconstruction algorithm E-iFEM. Meanwhile, the extracted geometric deformation is intended to qualify the advantages and limitations of the E-iFEM against existing or competing strategies.

**Figure 9:**
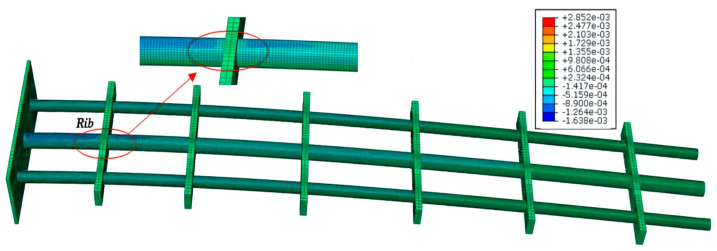
Detailed strain distribution information of ribs and beams.

To study the deformation characteristics of the airfoil frame, the stress and strain distribution information at the joint was calculated using the simulation software. It can be seen from the strain diagram that the strain state at the rib has an obvious change (see Figure 9). Therefore, the part with a large strain jump can be regarded as a rigid part.

According to the above preparation work, the complex aerostructure belongs to the typical rigid and elastic coupling model, while the traditional reconstruction scheme will increase the reconstruction error. Hence, several common evaluation indexes are introduced to quantify the effectiveness of the E-iFEM. The reconstructed nodal displacements were compared with the benchmark, and we obtain a relative root means square error (RRMSE):

(18) $$RRMSE=100\times RMSE/\left|MAX(disp^{ABAQUS})\right|\times \%,$$

It is noted that the RMSE was the following:

(19) $$RMSE=\sqrt{\frac{1}{n}\sum_{i=1}^{n}{\left(disp(x_{i})-disp{\left(x_{i}\right)}^{ABAQUS}\right)}^{2}},$$

Following the initial analysis, the airfoil frame is modeled using the coupled rigid-elastic inverse element with varying discretization. This preliminary execution aims to ensure the proper operation of the reconstruction algorithm. The detailed comparison data are in Table 2.

**Figure 10:**
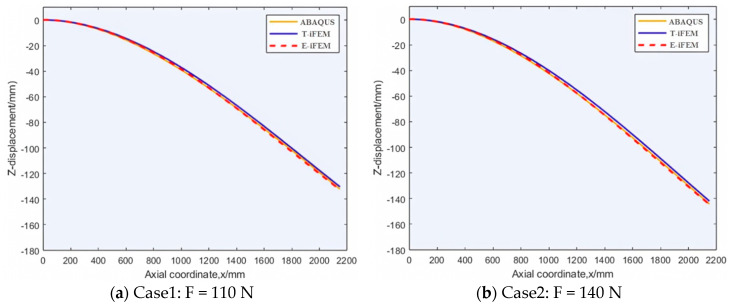

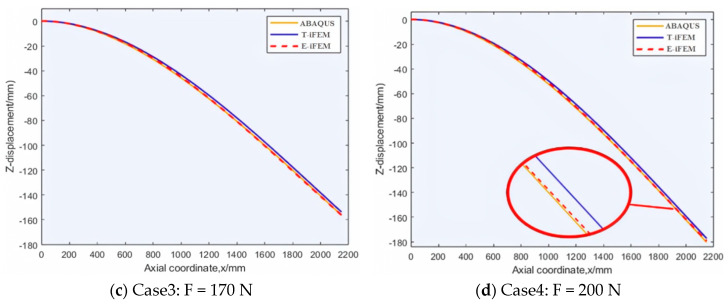
Comparison of ABAQUS, T−iFEM, and E−iFEM calculations.

For the elastic part of the structure, the multi-objective particle swarm optimization algorithm is used to arrange strain sensors, and the robustness and accuracy are taken as evaluation indexes. Therefore, the measured data of skin strain under different loads are extracted as the measurement system input, and the reconstructed displacement of the E-iFEM can be calculated. The reconstructed displacements using the proposed method, labeled “E-iFEM”, and the theoretical displacements from the simulation, labeled “ABAQUS”. In addition, the reconstructed displacement obtained by the traditional iFEM would be used for a comparison with the labeled “T-iFEM”. Meanwhile, four different end-concentrated loads were applied to the cantilever airfoil frame. The comparison between the reconstructed displacement obtained by the E-iFEM and the reference displacement is shown in Figure 10.

Based on Figure 10d, the yellow reference curve and red reconstruction curve E-iFEM basically coincide. Meanwhile, it can be accessed that the theoretical maximum deformation displacement in the z-direction is −182.187 mm. Compared with the T-iFEM, the reconstruction value of the proposed algorithm, the E-iFEM, is −181.659 mm. Hence, the defects of standard inverse finite elements in reconstructing complex wing structures will be analyzed. Since the standard inverse finite element takes the rib and elastomer as a whole unit, the reconstruction of the frame structure will produce errors at the first rib. Although the superiority of the iFEM in solving standard elastic structures has been demonstrated, the overall error will be accumulated with the increase in structural complexity.

The specific precision indicator of the enhanced iFEM under different loads is shown in Table 3, which describes the ME, the RRMSE, and the RMSE of displacement reconstruction error on eleven displacement-measuring points. The experimental results reveal that the reconstruction error RRMSE is no more than 0.6% within 185 mm of the deflection. Compared with the method that directly divides the airfoil frame into several elastic segments, the proposed E-iFEM can effectively solve the discontinuity problem at the theoretical and experimental levels. According to the above analysis, these results serve as an experimental validation of the E-iFEM element formulations, specifically for accurately modeling the contribution of discontinuous aerospace structures.

### 3.2. Simulation Study: Rectangular Section

**Figure 11:**
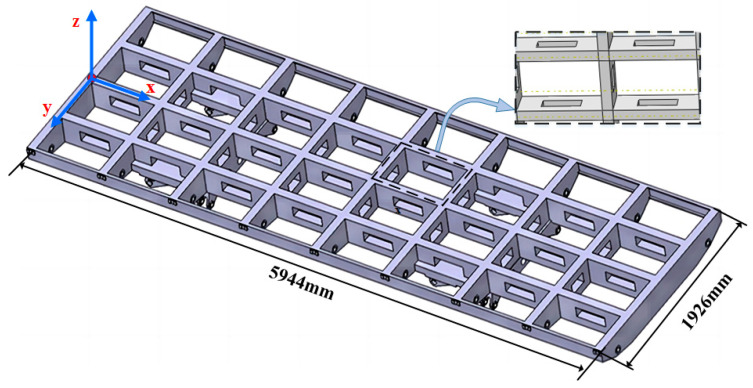
Simulation model for hollow rectangular beam.

Building on the success of previous results, we further used the combined antenna trusses to verify the reconstruction effectiveness of the E-iFEM. The antenna trusses with a more complex hollow rectangular cross-section in this simulation, as shown in Figure 11. The simulation model of the antenna truss is divided into 698,208 elements, including the beam and solid elements. The material parameters of the antenna trusses are chosen as follows: the component materials consist of structural steel (*E*_m_ = 200 GPa, *p*_m_ = 2700 kg/m^3^), and the coefficient *ν* is selected equal to 0.3. The full geometry dimensions of the simulation model are 5944 mm × 1926 mm. The interior of the main frame is proportionally arranged by three longitudinal beams and seven transverse beams, each spanning 653 mm. This comprehensive characterization encapsulates the intricacies of the antenna truss structure, emphasizing detailed specifications and dimensions, thus contributing to a thorough understanding of its finite element model.

**Figure 12:**
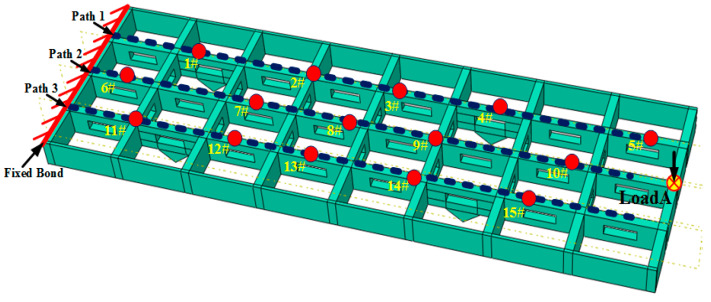
Layout of the deformation verification points and loading points.

It is worth noting that the 16 points are sent on the reinforced beam surface in Figure 12, among which points 1# to 15# are the verification points of deformation field inversion, and Load A is the loading point. Points 1#, 2#, 3#, 4#, and 5# are located on path 1. Points 6#, 7#, 8#, 9#, and 10# are all located in path 2, which is located in the inner middle line of the plate. Path 2 and Load A are located on the surface of the plate and symmetrically aligned with the complex section of the hollow rectangular. Points 11#, 12#, 13#, 14#, and 15# are all located in path 3, which is more concerned with the discontinuous part. In the finite element simulation analysis, the discontinuous structure is set to be unilaterally fixed. By applying equal Z-direction (perpendicular to the plate surface) loads at Load A, the structure can undergo bending conditions. According to the above working conditions, this paper mainly focuses on conducting the simulation validation of the displacement field reconstruction.

**Figure 13:**
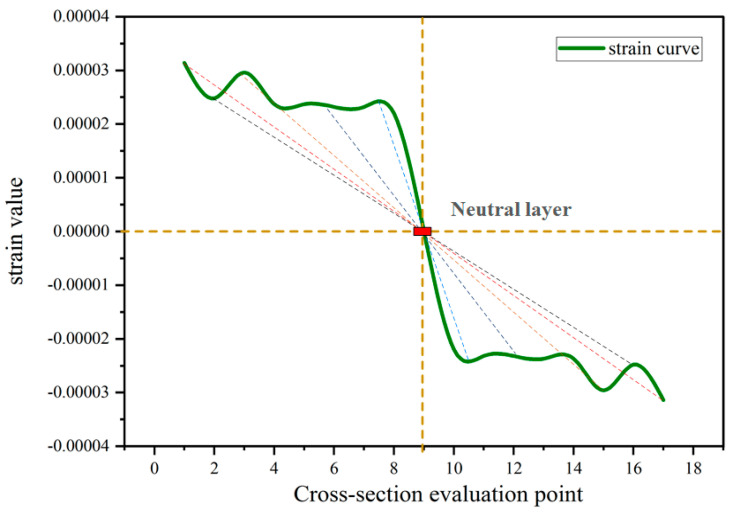
Simulation strain curves for the hollow rectangular section.

In conventional shell structures subjected to bending, the upper surfaces experience tensile strains, while the lower surfaces experience compressive strains. The strains have opposite signs, and their magnitudes tend to be the same. As strains are all zero at a position half the plate thickness away from the plate surface, this position is defined as the neutral layer. Hence, through the strategic placement of sensors on either the upper or lower surface of the measured plate, it becomes feasible to acquire the necessary data regarding both upper and lower surface strains. These data are crucial for reconstructing the strain field accurately using the E-iFEM. According to the extracted simulation data, the bending strain still has symmetry, as shown in Figure 13.

After determining the equivalent neutral layer position of the discontinuous hollow structure, the strain distribution function can be obtained, eliminating the need to use more grid cell division to calculate its strain distribution, which avoids the problem of singularity in the pseudo-stiffness matrix caused by excessive grid cell divisions and accelerates the convergence of the pseudo-stiffness matrix solution vector.

**Figure 14:**
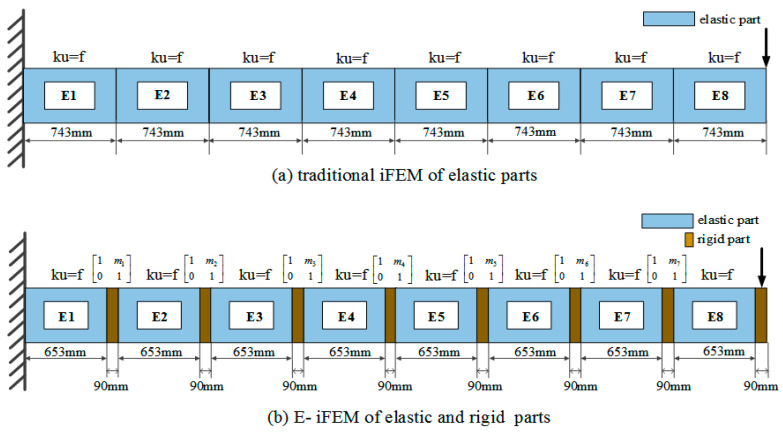
The inverse finite elements discretization of two methods.

To verify the reconstruction accuracy of the E-iFEM in complex variable bending structures, the hollow rectangular beam is divided into two reconstruction models and compared respectively: (a) The traditional iFEM of elastic part; (b) The E-iFEM of elastic and rigid parts. In the traditional iFEM, the hollow rectangular beam is evenly divided into eight inverse finite elements without considering the rib, as shown in Figure 14a. In the E-iFEM, the hollow rectangular beam is divided into eight inverse finite elements and eight rigid parts, as shown in Figure 14b. The rigid part can be considered to have high stiffness compared to the elastic parts. Upon obtaining information about the bending moment within a cross-section and the corresponding curvature, the unknown bending stiffness EI was formulated as follows:

(20) $$EI(x)=\frac{M(x)}{\kappa (x)}\;\;\kappa (x)=\frac{d\theta (x)}{dx},$$

The input parameters consist of strain data derived from strain-measuring points located along the elastic part of the hollow rectangular beam under various deflection angles. Subsequently, the reconstructed displacement values for both the E-iFEM and the T-iFEM are computed independently. Additionally, the assessment indicators are the same as in case 1. According to the verification point layout, the absolute error can be used to quantify the effectiveness of the reconstruction model.

**Figure 15:**
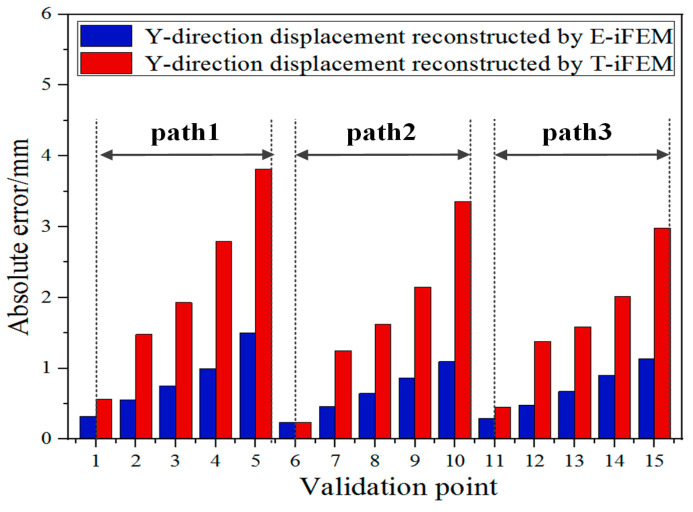
Comparison of displacement reconstruction errors between T-iFEM and E-iFEM.

As depicted in Figure 15, the inversion accuracy of the inverse finite element method based on elastic theory in Path1, Path2, and Path3 is lower than the E-iFEM. Utilizing the initial reconstructed displacement directly for the subsequent reconstruction of the complete deformation field may lead to the accumulation of inversion errors. This is because the displacement obtained by the iFEM in the rigid part is different from the actual displacement field. However, the E-iFEM based on rigid kinematics can reconstruct the displacement field without initial displacement error and guarantee reconstruction accuracy.

### 3.3. Experimental Calculation

**Figure 16:**
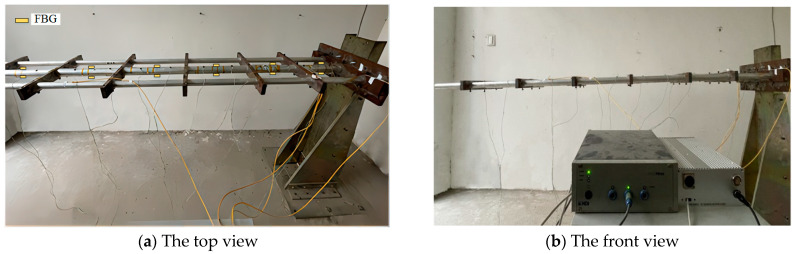
Experimental test platform for reconstruction algorithm E-iFEM.

In this section, the same-size test platform was manufactured to assess the reliability of the E-iFEM presented in this study. Three beams that are bound by rib plates are installed on the test platform, with one end locked by six bolt connections, as shown in Figure 16. Consistent with the simulation experimental model, the chief beam is a thin-walled aluminum alloy beam. Its length is 2150 mm, its outer radius is 20 mm, and its wall thickness is 4 mm. Given the substantial mass of the restraint base, the antenna plate can be conceptualized as an optimal cantilever structure, aligning well with the experimental specifications.

**Figure 17:**
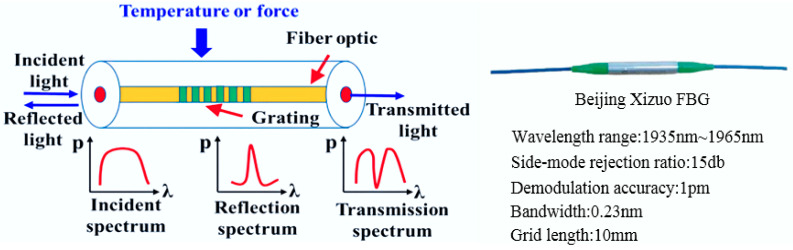
Experimental test platform for reconstruction algorithm E-iFEM.

Fiber Bragg grating is widely used in aerospace fields due to its small size, lightweight, and strong electrical insulation [28]. Therefore, Beijing Xizuo FBG is used as the contact strain sensor in this experiment, which relates the change in grating per unit length to the strain, and it is shown in Figure 17.

Simultaneously, the self-developed demodulation instrument is used to receive and interpret real-time fiber wavelength changes to determine the real-time strain. The third-party deformation data that can be used as reference standards are provided by the 3-D optical measurement device with an accuracy of 0.01 mm [NDI, Northern Digital Incorporated, Canada], which captures the measured deformation values of the discrete marked points on the test platform.

**Figure 18:**
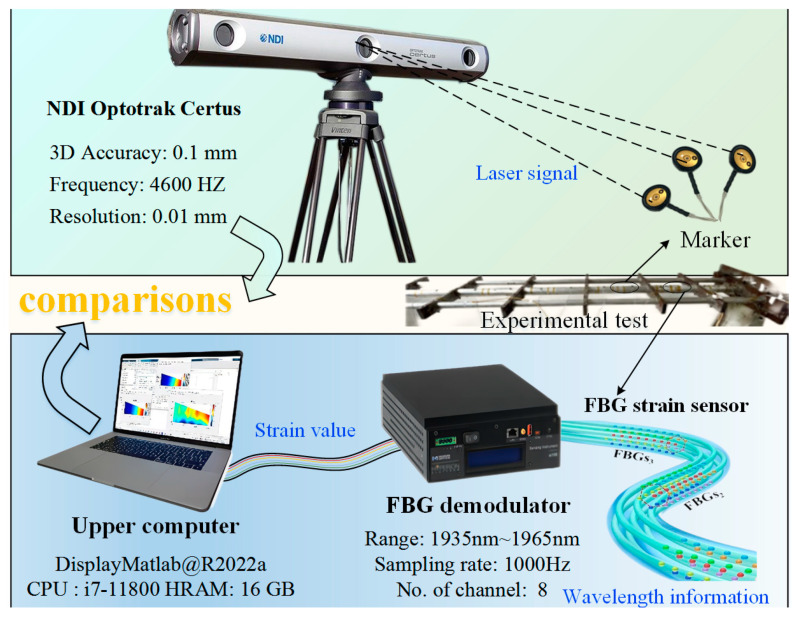
Experimental verification system.

First of all, when the end of the chief beam is subjected to elastic deformation under a concentrated load, the demodulator calculates and saves the corresponding strain values by reading the change in wavelength on the preset FBG. Then, the reconstructed deformation can be obtained by inputting the strain values into the deformation reconstruction algorithm. Subsequently, the third-party monitoring equipment from NDI is used to read the deformation at the calibration point and record the actual measurement data. The connections between the components in the experimental platform are shown in Figure 18. The coordinates of the position sensor are shown in Table 4, which is also the paste position of the actual mark point. The scattered infrared of the marker was obtained by the three-eye camera lens, and the deformation information can be displayed by computer software.

**Figure 19:**
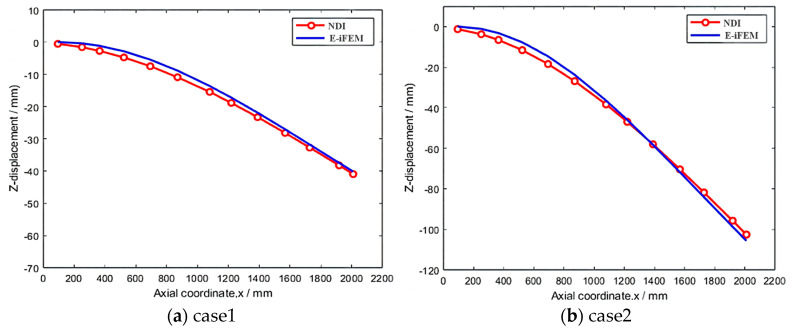
Comparison of reconstructed and measured displacements under different loads.

In line with the above steps, different concentrated loads are applied at the end of the main beam; the deformation information is based on NDI and the proposed algorithm. Figure 19 shows the distribution of reconstruction and measurement deformation along the x-axis. Additionally, the detailed values of the E-iFEM and from NDI in the two successive load steps, along with the evaluation index, are summarized in Table 4.

The evaluation index of the E-iFEM and NDI are counted in Table 5. The external concentrated load conditions include the small load of 50 N and the large load of 180 N, and the maximum deformation of the z-axis in the main deformation direction is −102.62 mm. Meanwhile, its reconstruction accuracy RMSE is 2.63 mm, and the RRMSE is 2.57%.

It should be noted that the iFEM was combined with rigid body kinematics to realize the real-time deformation monitoring of complex structures, has not yet been described in the available literature. Therefore, the reconstruction method proposed in this paper can accurately and stably sense the shape deformation of the aerospace structure in the physical experiment.

## 4. Conclusions

In this paper, a novel reconstruction algorithm for the shape and strain sensing of complex aerospace structures is presented. The primary innovation of the E-iFEM lies in its flexible combination of rigid kinematics with the classical iFEM for discretizing the reconfiguration model into rigid parts and elastic parts. The rigid parts provide geometric constraints, and the elastic parts are reconstructed with the standard iFEM. The proposed method retains the advantageous features of the iFEM, monitoring full-field structural behavior based solely on strain sensor data without requiring external load and material information. Compared to the iFEM, the E-iFEM is more suitable for smart aerospace structures as it can further reduce the number of sensors while maintaining accuracy.

Through a series of numerical simulations and an experiment conducted on discontinuous aerospace structure, the accuracy, effectiveness, and practicality of the E-iFEM method are demonstrated.

Numerical analyses indicate that utilizing the calculated rigid boundary enables efficient reconstruction of the deformation of the typical and hollow sections. Experimentally validated results reveal that the maximum RMSE is less than 3 mm from the E-iFEM and is directly measured by NDI. As expected, the proposed reconstruction deformation algorithm accurately predicts strain and deformation along the length of the wing structure with a small number of sensors. However, the experimental subject of this study was limited to linear deformations without expanding to nonlinear deformations.

## Figures and Tables

**Table 1:** Material type and mechanical parameters.

Component	Young’s Modulus	Poisson Ratio	Density
Rib	E = 210 Gpa	v = 0.33	P3 = 7850 kg/m^3^
girder beam	E = 70 Gpa	v = 0.33	P = 2712 kg/m^3^

**Table 2:** The Comparison under Two Loading Conditions in model-1.

Axial Coordinate /mm	110 N			200 N		
	ABAQUS	E-iFEM	T-iFEM	ABAQUS	E-iFEM	T-iFEM
200	−2.002	−1.755	−1.718	−2.692	−2.366	−2.330
400	−7.321	−6.842	−6.618	−9.890	−9.239	−8.991
600	−15.515	−14.947	−14.332	−21.032	−20.217	−19.503
800	−26.122	−25.456	−24.494	−35.540	−34.493	−33.390
1000	−38.676	−38.182	−36.734	−52.828	−51.831	−50.172
1200	−52.784	−52.603	−50.687	−72.387	−71.556	−69.374
1400	−68.026	−68.288	−65.986	−93.649	−93.099	−90.517
1600	−84.144	−84.846	−82.264	−116.254	−115.959	−113.123
1800	−100.832	−101.834	−99.151	−139.754	−139.553	−136.715
2000	−117.918	−118.858	−116.283	−163.869	−163.373	−160.814
2150	−130.872	−131.201	−129.070	−182.187	−181.659	−178.936

**Table 3:** Precision comparison of different evaluation indexes.

Sample	ME (mm)	RMSE (mm)	RRMSE (%)
Case1	1.047	0.603	0.46
Case2	0.964	0.561	0.52
Case3	0.856	0.719	0.53
Case4	1.001	0.673	0.37

**Table 4:** The geometric coordinates of the evaluate point.

Axial Coordinate /mm	NDI		E-iFEM	
	50 N	180 N	50 N	180 N
93.59	−0.54	−1.17	−0.05	0.08
250.13	−1.58	−3.72	−0.58	−1.04
364.51	−2.74	−6.53	−1.73	−3.13
522.23	−4.77	−11.56	−4.26	−7.67
693.57	−7.49	−18.42	−8.13	−14.62
870.39	−10.90	−26.88	−13.22	−23.73
1078.93	−15.47	−38.35	−20.34	−36.45
1219.57	−18.88	−46.95	−25.71	−45.99
1390.40	−23.30	−58.08	−32.67	−58.32
1568.87	−28.20	−70.40	−40.31	−71.80
1728.54	−32.75	−81.82	−47.34	−84.13
1919.15	−38.26	−95.77	−55.74	−98.78
2010.58	−40.98	−102.62	−59.73	−105.68

**Table 5:** Precision comparison for Load case 1 and Load case 2.

Sample	ME (mm)	RMSE (mm)	RRMSE (%)
50 N	2.05	1.46	3.56
180 N	3.89	2.63	2.57

## Data Availability

Data are contained within the article.
